# New Lyssavirus Genotype from the Lesser Mouse-eared Bat (*Myotis blythi*), Kyrghyzstan

**DOI:** 10.3201/eid0903.020252

**Published:** 2003-03

**Authors:** Yohko T. Arai, Ivan V. Kuzmin, Yosuke Kameoka, Alexandr D. Botvinkin

**Affiliations:** * National Institute of Infectious Diseases, Tokyo, Japan; † Institute for Natural Foci Infections, Omsk, Russia; ‡ Antiplague Research Institute of Siberia and the Far East, Irkutsk, Russia

**Keywords:** Lyssavirus, genotype, N gene, phylogenetic analysis, bat, central Asia, research

## Abstract

The Aravan virus was isolated from a Lesser Mouse-eared Bat *(Myotis blythi)* in the Osh region of Kyrghyzstan, central Asia, in 1991. We determined the complete sequence of the nucleoprotein (N) gene and compared it with those of 26 representative lyssaviruses obtained from databases. The Aravan virus was distinguished from seven distinct genotypes on the basis of nucleotide and amino acid identity. Phylogenetic analysis based on both nucleotide and amino acid sequences showed that the Aravan virus was more closely related to genotypes 4, 5, and—to a lesser extent—6, which circulates among insectivorus bats in Europe and Africa. The Aravan virus does not belong to any of the seven known genotypes of lyssaviruses, namely, rabies, Lagos bat, Mokola, and Duvenhage viruses and European bat lyssavirus 1, European bat lyssavirus 2, and Australian bat lyssavirus. Based on these data, we propose a new genotype for the *Lyssavirus* genus.

The *Lyssavirus* genus includes seven genotypes: rabies virus (RABV, genotype 1), Lagos bat virus (genotype 2), Mokola virus (genotype 3), Duvenhage virus (genotype 4), European bat lyssavirus 1 (EBLV-1, genotype 5), European bat lyssavirus 2 (EBLV-2, genotype 6), and Australian bat lyssavirus (ABLV, genotype 7) (*1*,*2*). Lagos bat virus was isolated from frugivorous bats (*Eidolon helvum*) in Nigeria in 1956 (*3*) and in 1974 from another bat *(Micropterus pusillus)* in the Central Africa Republic (*4*). Mokola virus was isolated from shrews (*Crocidura sp.*) and a child in Nigeria in 1968 (*5*,*6*), a girl in Nigeria in 1971 (*7*), and cats in Zimbabwe (*8*). Duvenhage virus was originally isolated from a human who died after being bitten by a bat in South Africa in 1970 (*9*) and from *Miniopterus sp.* bats in 1981 (*10*). EBLV-1 was isolated from bats (*Eptesicus serotinus)* in Germany in 1968 (*11*), in Poland in 1985 (*12*), in Denmark, Holland, and Spain in 1987, and in France in 1989 (*13*). Some isolates of EBLV-1 were obtained from bats in Ukraine and from one human case of bat origin in Russia in 1985 (*14*,*15*). EBLV-2 was isolated from a human in Finland in 1985 (*16*), and from bats in Holland, the Netherlands, Switzerland, and the U.K. EBLV-2 is mainly carried by bats of the *Myotis* genus (*Myotis dasycneme* and *M. daubentonii*) (*17*). ABLV was isolated from five species of flying fox bats, one species of an insectivorous bat, and two infected humans in 1996 (*1*,*18*,*19*).

Rabies viruses have been reported in Kazakhstan, central Asia (*20*). Terrestrial rabies viruses have been enzootic in all Central Asian countries and are mainly carried by dogs. Field rabies viruses have been isolated and characterized in Asia, specifically Pakistan, China, Indonesia, Thailand, the Philippines, Malaysia, India, and Sri Lanka (*21*–*26*). Isolation of lyssaviruses from bats has been reported only in India and Thailand; however, these viruses were reported as RABV (*27*,*28*). Recently, Arguin et al. detected neutralizing antibodies against ABLV in the serum of six bat species (*Mineopterus schreibersi, Taphozous melanopogan, Philetor brachypteus, Scotophilus kuhli, Pteropus hypomelanus,* and *Rousettus amplexicaudatus*) in the Philippines (*29*).

Aravan virus was originally isolated from the brain of a lesser mouse-eared bat (*Myotis blythi*) in Kyrghyzstan in 1991. The antigenic profile of the virus was analyzed by using two panels of antinucleocapsid (N) gene monoclonal antibodies developed at the Wistar Institute of Anatomy and Biology (USA) and the Central Veterinary Laboratory of Great Britain (Weybridge, U.K.) (*30*–*32*). These results demonstrated that the virus differed from rabies and serotypes 2 (Lagos bat virus), 3 (Mokola virus), 4 (Duvenhage virus), 5 (EBLV-1), and 6 (EBLV-2). Furthermore, 386 nucleotides (nt) of the N gene were determined from reverse transcription-polymerase chain reach (RT-PCR) product. Phylogenetic analysis suggested that the Aravan virus did not belong to the rabies virus group (*33*). In the present study, we determined the entire coding region of the N protein of Aravan virus and evaluated the phylogenetic relationships with other members of the *Lyssavirus* genus.

## Materials and Methods

### Viruses

Aravan virus was isolated from the brain of one lesser mouse-eared bat (*Myotis blythi*) during a survey of 269 bats collected in the Osh region of Kyrghyzstan from 1988 to 1992 (*30*,*32*). A direct fluorescent antibody test was conducted. Aravan virus–infected mouse brains were impressed on glass slides, air-dried, and fixed with acetone. To detect the lyssavirus antigen, specimens were stained with fluorescein isothiocyanate (FITC)–labeled anti-rabies globulin (BBL, Cockeysville, MD) or FITC-labeled anti-rabies monoclonal globulin (Centocor Inc., Malvern, PA). FITC-labeled anti-nucleoprotein monoclonal antibodies (NC-MAbs, W502) cross- reactive to lyssaviruses were also used (*19*).

### Amplification of Nucleoprotein cDNA and Direct Sequencing

Total RNA was extracted from virus-infected mouse brain emulsions with a commercial reagent (RNeasy Mini Kit, QIAGEN, Germany). cDNA was obtained with a T-Primed First-Strand kit (Amersharm Biosciences Corporation, Piscataway, NJ). PCR amplification and sequencing of the N gene were performed by using the sense primer AraN-S01 (5´-ATGTACCACCTCTACAATGG-3´, nt 55–74) and an antisense primer AraNC-1400 (5´-TCATGCTCAATTGTAAAAC-3´, nt 1456–1474). The cDNA template (2 μL) was amplified by using primers (AraN-S01 and AraNC-1400), according to the manufacturer’s instruction (Super Taq Premix Kit, Sawady Technology, Tokyo, Japan). PCR reactions were incubated at 94°C for 2 min, subjected to 40 cycles of 94°C for 30 s, 48° for 20 s, and 68°C for 2 min, and a final extension at 68°C for 7 min in a DNA thermal cycler (GeneAmpPCR System 9700 Applied Biosystems, Perkin-Elmer Corporation, Japan) (*24*,*25*). PCR products were purified by using a commercial kit (QIAquick PCR Purification Kit, QIAGEN). The sequences of the purified DNA products were determined on an automated sequencer (ABI model 310, Applied Biosystems, Foster City, CA) by using a PRIMS Ready Reaction Dyedeoxy Terminator Cycle Sequencing Kit (Applied Biosystems).

### Phylogenetic Analysis

The 1350-nt and the deduced 450 amino acid (aa) sequences of the N gene of the Aravan virus were aligned with 26 lyssaviruses by using Clustal W program (*34*). A phylogenetic tree was constructed with the computer software **MEGA 2** (*35*). Pairwise evolutionary nucleotide distances, including both transitions and transversions, were estimated according to Kimura’s two-parameter method. Phylogenetic trees were constructed by the neighbor-joining method with 1,000 replicates to generate bootstrap probabilities at each node (*36*).

## Results and Discussion

### Direct Fluorescent Antibody Assay

The three strains used in this study reacted against the Aravan virus infected mouse brain impressions. Fluorescence showed more scattered inclusions than those of the challenge virus standard in the acetone-fixed mouse brain smear (data not shown). The results confirmed that the Aravan virus is a lyssavirus.

### Nucleotide and Deduced Amino Acid Sequence Identities among the Aravan Virus and Other Lyssaviruses

The 1350-nt and the deduced 450 aa sequences of the Aravan virus were compared with 26 representative lyssaviruses belonging to seven genotypes (Table 1). We selected 16 representative rabies variants from the eight diverse groups, including rabies variants from geographic areas of Asia near Kyrghyzstan and from bats and raccoons in North and South America (*25*,*37*). The nucleotide and amino acid sequence identities among all 27 lyssaviruses, including Aravan virus, were calculated. Then genotype 1 was represented by seven rabies viruses (SRL1032, 86118BRE, 1500AFS, 9218TCH, 8738THA, insectivorous bat/Chile, and PA R89), and genotypes 2, 3, 4, 5, 6, and 7 were represented by Lagos bat virus (8619NGA), Mokola virus (MOK/U22843), Duvenhage virus (86132AS), EBLV-1 (8918FRA), EBLV-2 (9007FIN), and ABLV (Balina/AF006497), respectively (Table 2). The nucleotide sequence identity of Aravan virus with the genotypes 4, 5, 6, and 7 was 77% to 78%; with genotype 1, 75% to 77%; and with genotypes 2 and 3, 72% to 74%. The most extensive nucleotide sequence differences among isolates of genotype 1 were between the raccoon isolate (PA R89) and the African and Asian isolates (82.8% to 82.9% identity). The Aravan virus demonstrated 92% aa sequence identity with genotypes 4, 5, and 7; 89% with genotype 6; and 81% to 85% with genotypes 2 and 3. The maximum variation of amino acid sequences within genotype 1 was exhibited between a vampire bat isolate from Brazil and an African isolate (93.1% to 93.3% identity). Genotype 4 (Duvenhage virus) was most closely related to genotype 5 (EBLV-1) with nucleotide and amino acid sequence identities of 79.8% and 93.3%, respectively. ABLV (genotype 7) was closely related to SRL1032 (genotype 1, Sri Lankan rabies virus) with a 93.1% aa sequence identity. These values were almost same as maximum variation of genotype 1. Based on our present data, we determined that isolates sharing <79.8% nt and 93.1% to 93.3% aa sequence identities belonged to different genotypes. In several studies, thresholds of <80% nt and 92% or 93% aa sequence identities warranted the proposal of a new genotype (*1*,*23*,*38*). Hence, the nucleotide and amino acid percentage identity values demonstrated that Aravan virus should be regarded as a new lyssavirus genotype.

**Table 1:** Lyssavirus isolates used in this study

Genotype^a^	Yr isolated	Virus (strain)	Country of isolation	Host	Accession no.		
1 (Rabies)	?	CTN	China	?	AF367863		
1 (Rabies)	1983	8738THA	Thailand	Human	U22653		
1 (Rabies)	?	?	India	?	AF374721		
1 (Rabies)	1996	SRL1032	Sri Lanka	Jackal	AB041964		
1 (Rabies)	1992	9218TCH	Chad	Dog	U22644		
1 (Rabies)	1988	9141RUS	Russia	Arctic fox	U22656		
1 (Rabies)	?	9196FX	Canada	Vulpes vulpes	L20676		
1 (Rabies)	1987	1500AFS	Rep.South Afr.	Yellow mongoose	U22628		
1 (Rabies)	1985	9142EST	Estonia	Racoon dog	U22476		
1 (Rabies)	1986	8681IRA	Iran	Dog	U22482		
1 (Rabies)	1985	86118BRE	Brazil	Vampire bat	U22479		
1 (Rabies)	1992	BBCAN	Canada	*Eptesicus fuscus*	AF351833		
1 (Rabies)	1992	MYCAN	Canada	*Myotis lucifugus*	AF351839		
1 (Rabies)	?	?	Chile	*Tadarida brasiliensis*	AF070450		
1 (Rabies)	1988	Insectivorous Bat	Chile	*Insectivorous bat*	AF351850		
1 (Rabies)	1989	PA R89	USA	Raccoon	U27221		
2 (Lagos bat)	1958	8619NGA	Nigeria	*Eidolon helvum*	U22842		
3 (Mokola)	?	Y09762	?	?	Y09762		
3 (Mokola)	1981	MOK	Zimbabwe	Cat	U22843		
4 (Duvenhage)	1986	86132AS	Rep.South Africa	Human	U22848		
5 (EBLV-1)	1985	8615POL	Poland	*Eptesicus serotinus*	U22844		
5 (EBLV-1)	1989	8918FRA	France	*E. serotinus*	U22845		
6 (EBLV-2)	1986	9007FIN	Finland	Human	U22846		
6 (EBLV-2)	1986	9018HOL	Holand	*M. dasycneme*	U22847		
7 (ABLV	1996	Ballina	Australia	*Pteropid alecto*	AF006497		
7 (ABLV)	1996	Insectivorous isolate	Australia	Insectivorous bat	AF081020		
?	1991	Aravan	Kyrghystan	*M. blythi*	AB094438		

^a^EBLV-1, European bat lyssavirus 1; EBLV-2, European bat lyssavirus *2*; ABLV, Australian bat lyssavirus; MOK, strain name in Mokola virus.

**Table 2:** Comparison of nucleotide and deduced amino acid sequences of Aravan virus with other 13 lyssaviruses

Amino acid sequence identity (%)	Nucleotide sequence identity (%)																
			Genotype 1 (rabies virus)									Genotype 2	Genotype 3	Genotype 4	Genotype 5	Genotype 6	Genotype 7
	Aravan		SRL1032	U22479BRE	U22628AFS	U22644CHAD		U22653THA		AF351850	U27221	LBU22842	MKU22843	86132AS	8918FRA	9007FIN	AF006497
Aravan		100.0	75.6	76.0	76.2		74.8	75.9	77.0		76.2	74.3	72.4	78.2	77.9	77.2	76.9
SRL1032		90.9	100.0	84.7	85.9		86.9	86.8	86.6		84.1	74.2	70.2	73.9	75.6	74.7	78.0
U22479BRE		88.2	95.3	100.0	83.3		**83.0^a^**	83.3	89.9		83.7	73.8	69.6	73.8	75.3	74.2	77.2
U22628AFS		89.3	96.7	**93.3^b^**	100.0		85.6	83.5	83.6		83.2	73.3	70.4	73.9	75.3	74.6	78.0
U22644CHAD		88.7	96.9	**93.1 ^b^**	94.4		100.0	86.0	84.0		**82.9^a^**	73.0	69.0	73.1	75.0	74.8	77.2
U22653THA		89.8	97.1	94.2	95.1		95.3	100.0	84.2		**82.8^a^**	73.4	69.7	74.4	75.9	74.7	77.3
AF351850		90.0	97.1	95.8	95.8		94.9	95.6	100.0		86.1	73.5	70.1	74.3	75.0	74.8	77.4
U27221		89.6	95.8	93.6	94.2		93.8	94.9	95.1		100.0	73.1	70.2	74.2	74.6	75.7	77.1
LBU22842		84.7	82.9	81.8	81.1		80.4	81.8	83.1		82.2	100.0	74.8	73.4	74.4	72.5	72.6
MKU22843		80.9	78.2	77.6	77.3		76.2	77.6	78.4		77.8	84.4	100.0	71.6	69.9	69.2	71.0
86132AS		91.8	88.9	86.9	87.6		87.1	87.6	88.7		87.8	85.8	80.7	100.0	**79.8^c^**	75.9	77.0
8918FRA		92.0	88.9	86.7	87.8		87.8	88.4	88.2		88.2	83.8	79.1	**93.3^d^**	100.0	78.0	76.9
9007FIN		88.9	88.0	86.2	87.1		86.9	88.0	87.3		87.3	79.1	76.2	86.2	88.0	100.0	77.2
AF006497		92.0	**93.1^d^**	91.1	91.6		91.1	91.8	91.8		91.3	82.0	79.8	90.0	89.8	87.8	100.0

^a^The values were shown as maximum variation of nucleotide sequence identities (%) within genotype 1. ^b^The values were shown as maximum variation of amino acid sequence identities (%) within genotype 1. ^c^Thresholds of nucleotide sequence identities % as different genotypes. ^d^Thresholds of amino acid sequence identities % as different genotypes.

### Phylogenetic Analysis

A phylogenetic tree of 27 lyssaviruses, including the Aravan virus, based on the 1350-nt sequence of the N gene was constructed by using the vesicular stomatitis Indiana virus (VSIV, tsW16B/U13898) as an outgroup (Figure, a). The lyssaviruses divided into two groups: one group consisted of genotypes 2 and 3, and the other consisted of genotypes 1, 4, 5, 6, 7, and the Aravan virus. The latter group was divided into six distinct clusters corresponding to genotypes 1, 7, 6, and 5 (high bootstrap values of 98%, 99%, 100%, and 100%, respectively), then Aravan virus and genotype 4. Moreover, the Aravan virus clustered with genotypes 4, 5, and 6 (low bootstrap value of 59%). Duvenhage virus (genotype 4) and EBLV-1 (genotype 5) formed the same cluster (high bootstrap value of 91%), and are therefore closely related. The Aravan virus occupied the phylogenetic position between genotype 6 and the cluster of genotypes 4 and 5. We also constructed a phylogenetic tree based on the deduced 450-aa sequences of the N gene (Figure, b). Similar to the nucleotide data, the amino acid sequences divided into two large groups and further subdivided into eight groups. One group consisted of genotypes 2 and 3 (bootstrap value of 89%), and the other group consisted of genotypes 1, 7, 6, 4, and 5, and the Aravan virus (high bootstrap value of 100%). The latter group had three distinct clusters corresponding to genotypes 1, 7, and 6 (high bootstrap values of 100%, 99%, and 100%, respectively), genotypes 4 and 5 (same cluster with a high bootstrap value of 98%), and the Aravan virus. The Aravan virus did not group with any other genotypes and is located at a position close to the cluster of genotypes 4 and 5 (bootstrap value of 66%).

**Figure:**
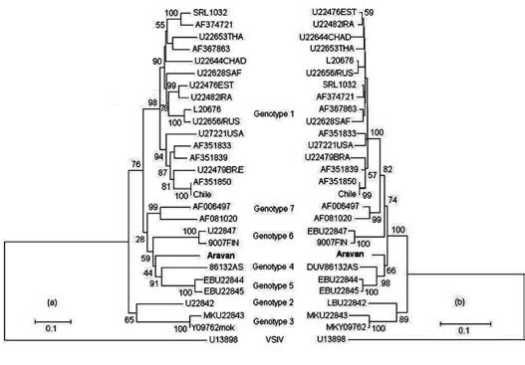
Rooted phylogenetic tree showing genetic relationships among Aravan virus and 26 lyssaviruses. Phylogenetic relationships were determined by comparing the 1350-nucleotide sequences of the nucleoprotein (N) gene (a) and the deduced 450-amino-acid sequences (b) by the neighbor-joining method (36). The sequences used were those of genotypes 1, 2, 3, 4, 5, 6, and 7 shown in Table 1 by using vesicular stomatitis Indiana virus (VSIV) as an outgroup (tsW16B/U13898).

These results, along with those in Table 2 and the Figure, suggest that the Aravan virus does not belong to any of the seven Lyssavirus genotypes (rabies, Lagos bat, Mokola, Duvenhage, EBLV-1, EBLV-2, and ABLV). Thus, we propose that the Aravan virus forms an independent cluster and is a new member of the *Lyssavirus* genus.

In this article, we have reported the first lyssavirus distinct from rabies virus originating on the Asian continent. The Aravan virus was more closely related to genotypes 4, 5, and, to a lesser extent, 6, which circulates among insectivorus bats in Europe and Africa. The lesser mouse-eared bat, from which the Aravan virus was isolated, is widely distributed in northern Africa, the Mediterranean, southern Europe, Crimea, Caucasus, Palestine, southwest Asia, and parts of central and eastern Asia. This information should be considered in the discussion of lyssavirus classification and evolution, as it suggests the possibility of a broader geographic distribution of the Aravan virus. We have no information about human rabies caused by bat exposure from central Asia, and rabies surveillance in this area is not known well. Based on this information and the virus’ misdiagnoses as rabies, we consider that transmission of Aravan virus to humans is possible. Indeed, this finding stimulates interest in new genotypes of lyssaviruses and is important from the viewpoint of public health, necessitating further lyssavirus surveillance of bats on the Asian continent.
